# Uncovering the molecular networks of ferroptosis in the pathogenesis of type 2 diabetes and its complications: a multi-omics investigation

**DOI:** 10.1186/s10020-024-01045-w

**Published:** 2024-12-23

**Authors:** Changqing Dong, Wuda Huoshen, Yunfeng Bai, Jiaona Liu, Bing Li, Yucan Guan, Ping Luo

**Affiliations:** 1 Department of Nephrology, National Key Laboratory of Diabetes, The Second Hospital of Jilin University, No. 991 Yatai Street, Nanguan District, Changchun, Jilin China; 2 Department of Nephrology, First Medical Center of Chinese PLA General Hospital, National Key Laboratory of Kidney Diseases, National Clinical Research Center for Kidney Diseases, Beijing Key Laboratory of Kidney Diseases Research, Beijing, China; 3 School of Stomatology, Southwest Medical University, Luzhou, Sichuan China

**Keywords:** Diabetic complication, Ferroptosis, Mendelian randomization, Multi-omics, NAFLD, Non-invasive diagnosis, Type 2 diabetes

## Abstract

**Background:**

Diabetes is a multi-factorial disorder and related complications constitute one of the principal causes of global mortality and disability. The role of ferroptosis in diabetes and its complications is intricate and significant. This study endeavors to disclose the role of ferroptosis in the aforementioned diseases from multiple perspectives through multi-omics.

**Methods:**

We performed genetic correlation analyses via the Linkage Disequilibrium Score and High-Definition Likelihood approaches for type 2 diabetes (T2D) and its complications. The data concerning the expression of ferroptosis-related genes (FRGs) were obtained from the meta-analysis of studies on gene expression and protein abundance. Mendelian randomization analyses and cross-validation were implemented using the discovery cohort, replication cohort, and imaging genomics cohort of T2D and its complications. Moreover, we conducted colocalization analyses on T2D and tissue-specific single-cell RNA sequencing investigations on the complications to complement the results.

**Results:**

Genetic association analysis indicated that the selected datasets could be incorporated into a secondary analysis of T2D complications. In the primary analysis, six FRGs (CDKN1A, ENO3, FURIN, RARRES2, TYRO3, and YTHDC2) were found to be positively associated with T2D risk. Conversely, eight FRGs (ARNTL, CAMKK2, CTSB, FADS2, KDM5A, MEG3, SREBF1, and STAT3) were inversely associated with T2D risk. The 14 FRGs were included in the secondary analysis. Within the FRGs, which received full support from both the discovery and replication cohorts, and were further validated by imaging genomics, higher levels of CDKN1A were positively associated with DKD risk. Higher levels of CAMKK2 and KDM5A were associated with a decreased risk of DKD. For DCM, higher levels of CTSB were positively associated with DCM risk. And genetically predicted higher levels of ARNTL and SREBF1 were associated with a decreased risk of NAFLD. Finally, we validated the tissue-specific expression of each complication with scRNA-seq datasets.

**Conclusions:**

This study identified FRGs in relation to T2D and its complications, which may enhance the understanding of the pathogenic mechanisms of their development. Meanwhile, it offers cross-validation for imaging genomics and further indicates the direction for non-invasive diagnosis.

**Graphical Abstract:**

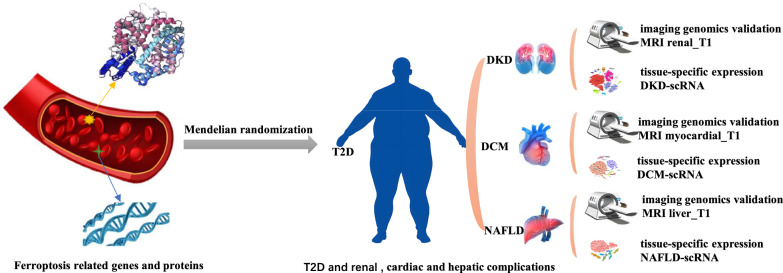

**Supplementary Information:**

The online version contains supplementary material available at 10.1186/s10020-024-01045-w.

## Background

Global Burden of Diseases reveals that the burden of diabetes has witnessed a remarkable increase, which is projected that 693 million adults will be afflicted by 2045 (Ong et al. 2023). Among them, type 2 diabetes (T2D), encompassing various pathophysiological states, currently constitutes approximately 90–95% of all diabetic patients, while diverse forms of T2D all possess significant genetic factors (Cole and Florez 2020). Therefore, from a clinical perspective, this study primarily focuses on T2D.

The various complications of T2D develop further damage to numerous systems, and these complications constitute the predominant problem in healthcare. The renal, cardiac and hepatic complications were included in the secondary analysis in this study as they are the main causes of disease progression and mortality in diabetic patients (Liu et al. 2024). Diabetic kidney disease (DKD) constitutes a severe microvascular of T2D. Approximately 30–40% of patients with T2D concurrently experience DKD, which leading to manifestations such as frailty, diminished quality of life, end-stage renal disease. Diabetic cardiomyopathy (DCM) constitutes a notable complication for patients with T2D and stands as the primary cause of heart failure in individuals afflicted with T2D (Tan et al. 2020). Non-alcoholic fatty liver disease (NAFLD) which may progress to cirrhosis and hepatocellular carcinoma, affecting approximately 25% of the global population. There is mutual interference between NAFLD and T2D. Statistics show that the prevalence of NAFLD in patients with T2D (76%) is significantly higher (Younossi et al. 2016).

Ferroptosis has been recognized as a form of regulated cell death recently, which involves gene and protein regulation. And is associated with the accumulation of abnormal lipid reactive oxygen species (ROS), ultimately leading to oxidative stress and triggering cell death (Yan et al. 2021). Ferroptosis affect pancreatic β cells of diabetic patients through multiple pathways such as iron metabolism, lipid and amino acid metabolism, mitochondrial metabolism, thereby influencing the occurrence and development of T2D (Krümmel et al. 2021). Ferroptosis also causes damage to multiple tissues and organs as a result of peroxidative accumulation of polyunsaturated fatty acids (PUFAs) and uncontrolled REDOX reactions (Tong et al. 2022). Many recent studies also indicate that targeting ferroptosis may be an effective therapeutic strategy for diabetes and its complications (Liu et al. 2024).

Mendelian randomization (MR) analysis employs genetic variations as instrumental variables to enhance the inference of causal associations between exposures and outcomes. The continuous proliferation of large-scale genome-wide association studies (GWAS) and molecular quantitative trait loci (QTL) data enables us to explore the causal relationship between ferroptosis regulation and T2D as well as its complications at multi-omics levels with MR approach (Skrivankova et al. 2021).

The validation of radiomics and imaging genomics was introduced to assist us in addressing the challenges posed by biopsy complications (such as major hemorrhage or arrhythmia). Based on the trait of the close correlation between T1 time and glycolipid metabolism and iron homeostasis (Nauffal et al. 2024), We chose an imaging genomics investigation on T1 time as an additional validation for the analyses of DKD, DCM, and NAFLD. It also offers the potential for the subsequent studies of the complications, with the aim of prognostic evaluations to reduce the large-scale mortality rate caused by tissue fibrosis of kidney, myocardium, and liver (Nauffal et al. 2024). Simultaneously establishing a solid foundation for the non-invasive diagnosis and prognosis determination of diseases in imaging genomics (Singh et al. 2020; Puntmann et al. 2016; Parisinos et al. 2020).

In this study, we adopted the methods of Linkage disequilibrium score (LDSC) and High-Definition Likelihood (HDL) to evaluate the shared genetic architecture between T2D and its complications. Subsequently, with the aid of large-scale eQTLs and pQTLs, supplemented by colocalization analysis, along with replication cohorts, imaging genomics and scRNA transcriptomics, we conducted multiple perspectives and mutually verified explorations.

## Methods

### Study design

**Fig. 1:**
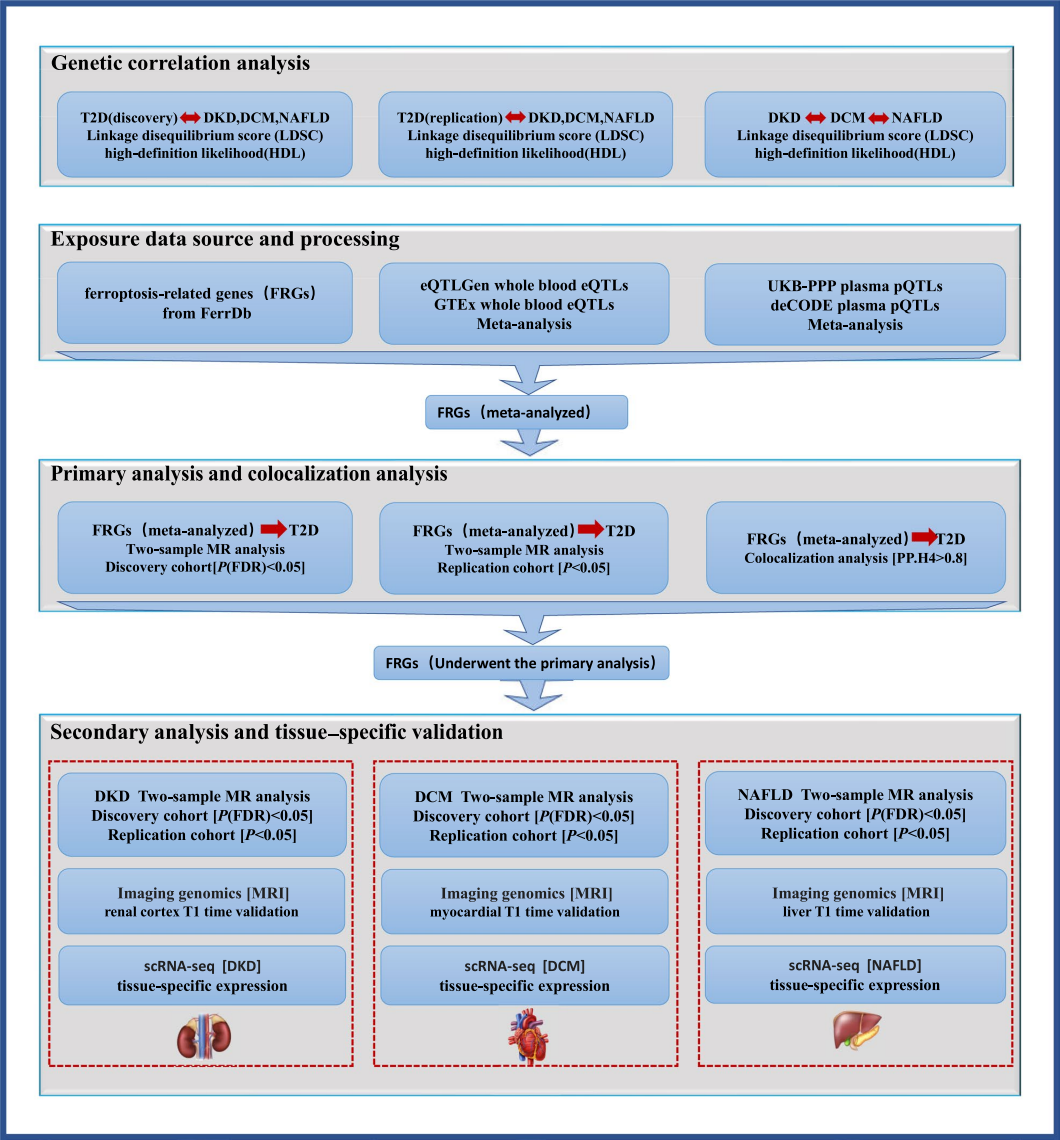
Summary of the study design

This study constitutes a secondary analysis of publicly accessible data. In accordance with the protocol of each original GWAS datasets, all participants provided informed consent, and all ethical approvals for the GWAS were acquired by the original authors of the GWAS. The overall summary the design of this study is shown in Fig. 1.

### Genetic correlation analysis

Currently, LDSC (Bulik-Sullivan et al. 2015) and HDL (Ning et al. 2020) are commonly utilized to evaluate the genetic structure shared between two complex phenotypes. To assess the representativeness of the datasets with greater accuracy, we conducted genetic correlation analyses using both LDSC and HDL methods. Specifically, we performed these analyses on the discovery and replication cohorts of T2D as well as the discovery and replication cohorts of the complications to be included in the secondary analyses.

### Exposure data source and processing

To determine the characteristics of ferroptosis, we retrieved ferroptosis related genes, which verified experimentally in FerrDb (Zhou et al. 2023). Gene expression data derived from expression quantitative trait locus (eQTL) studies of eQTLGen Consortium and GTEx (whole blood) (Võsa et al. 2021; GTEx Consortium 2020). Protein abundance data derived from two largest cohorts of plasma protein studies were extracted, namely deCODE protein quantitative trait loci (pQTL) study carried out by Ferkingstad et al. on 35,559 Icelanders and the UKB-PPP protein study cohort (46,218 data with British or Irish ancestry) (Eldjarn et al. 2023). In this study, regarding the expression data acquired above, a meta-analysis (MeCS) (Qi et al. 2018) was carried out between the eQTLGen Consortium and GTEx v8 whole blood, with the aim of eliminating heterogeneity and biases resulting from the analysis. Likewise, meta-analyses were implemented for the two protein abundance study datasets as mentioned, namely deCODE and UKB-PPP. Given that the proteins level is closer to the disease and has a better reflection of the disease status, the intersected data of the meta-analyzed protein datasets and the ferroptosis-related genes were selected. In case of missing data in the current proteomics study, the meta-analyzed gene expression data was employed as a substitute. Here, the Cis-region SNPs of proteins or genes data were defined as SNPs within ± 1000 kb from the start and end of the gene encoding the protein, and 5E-8, R^2^ < 0.1 (based on reference data files created from Phase 3 of 1,000 Europeans) and F-statistic > 10 were set as the criteria for screening exposure instrumental variables. Finally, 83 proteins abundance levels and 145 genes expression levels related to ferroptosis were obtained, which are collectively referred to as ferroptosis-related genes (FRGs) in this study.

### Outcome data source

Discovery cohort datasets: The dataset of T2D originates from the DIAGRAM consortium, encompassing a meta-analysis of 122 studies (Mahajan et al. 2022). DKD dataset derived from a GWAS meta-analyses, and the “All vs Ctrl” phenotype was chosen as the discovery cohort for DKD. DCM dataset originates from a study diagnosed with cardiomyopathy by on the diagnostic codes (ICD10) from UK Biobank. NAFLD dataset was a study which based on the diagnostic codes (ICD10) recommended in the guidelines for GWAS (Table S1).

Replication cohort datasets: The data of T2D, diabetic nephropathy, cardiomyopathy, and NAFLD from the FinnGen study were respectively selected as the replication cohorts (Kurki et al. 2023).

Imaging genomics datasets: A machine learning model for magnetic resonance imaging of 43,881 UK Biobank participants was selected to quantify the alterations in tissue-specific T1 time in association with diseases. The summary data of T1 GWAS for the kidneys, heart, and liver in this study were respectively adopted as the cross-validation datasets (Table S1) (Nauffal et al. 2024).

### Primary analysis and colocalization

MR analysis was conducted using the TwoSampleMR package in R (V4.3.2). The IVW (inverse variance-weighted) method was employed to estimate the odds ratio (OR) and corresponding confidence interval (CI) associated with the FRGs for each outcome to obtain the most precise and powerful estimates (Pierce and Burgess 2013). FRGs with less than 3 SNPs in the analysis were excluded as they could not complete the MR-Egger intercept test. Depending on heterogeneity, either a random-effects model or a fixed-effect model was utilized. MR-Egger regression was carried out to test potential pleiotropy in the association between FRGs and T2D. FRGs with the same directionality in the five default MR methods and no pleiotropy (*P* > 0.05) were retained. The results of discovery cohort analysis were corrected by false discovery rate (FDR) in both the primary and secondary analyses, and *P* (FDR) < 0.05 were determined to be significantly correlated. The replication cohort and imaging genomics cohort were successfully validated with *P* < 0.05.

We perform Bayesian colocalization analysis to assess whether FRGs and T2D were consistent with a shared causal variant based on the “coloc” package with default parameters. In this study we included SNPs within ± 100 kb of the significant variation sites for each FRGs cis-region, and the posterior probability for PP.H4 ≥ 0.8 was considered to have strong colocalization support for both signals.

### Secondary analysis and tissue-specific validation

The discovery cohort and replication cohort in the MR analysis of FRGs and T2D were highly significant, and genes with shared causal variation (PP.H4) ≥ 0.8 were utilized for the subsequent analysis of renal, cardiac and hepatic complications. In secondary analyses, genes supported by the discovery cohort, replication cohort, and imaging genomics cohort were identified as exerting significant effects on tissue complications.

To further investigate the tissue-specific expression pattern of ferroptosis related genes, we chose GSE195460, GSE213337, and GSE174748, and regarded them as single-cell transcriptome validation sets for DKD, DCM, and NAFLD respectively. Given the challenge of obtaining human specimens of DCM, in this study, the GSE213337 of mouse DCM was utilized as a secondary analysis for complications of T2D. The datasets were respectively processed for quality control, standardization, dimensionality reduction, and annotation using Seurat packages (Version 4.4). The tSNE dimension reduction was applied, and the cell clusters of tSNE were annotated according to the Marker Genes (Abdulla et al. 2023).

## Results

### Genetic correlation analysis

**Fig. 2:**
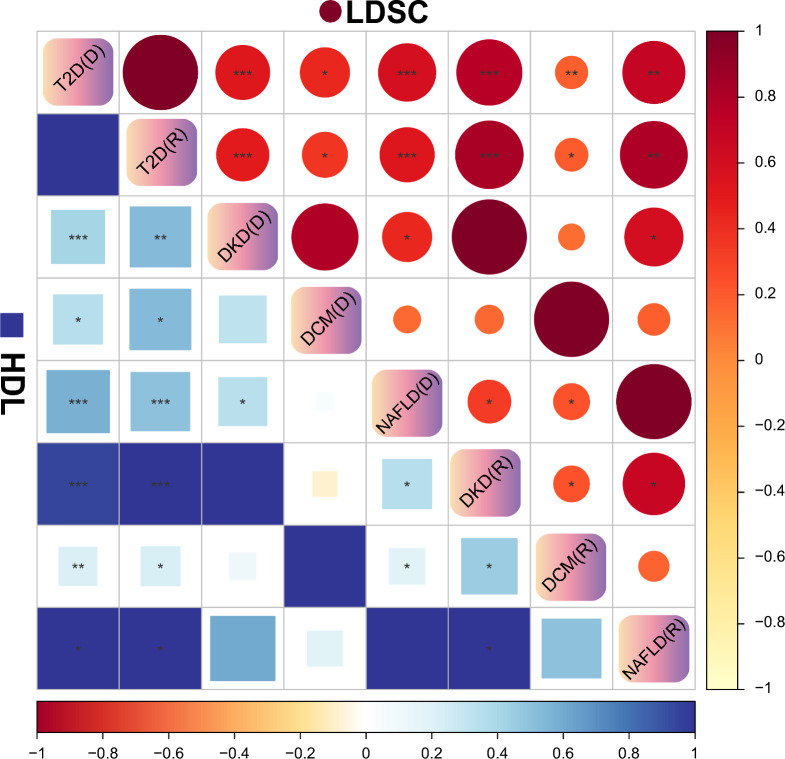
Heat map of genetic correlations between T2D and datasets included in secondary analyses. *LDSC* linkage disequilibrium score regression, *HDL*, high-definition likelihood; *T2D*, type 2 diabetes, *DKD* Diabetic kidney disease, *DCM* Diabetic cardiomyopathy, *NAFLD* Non-alcoholic fatty liver disease; (D), datasets of discovery cohort; (R), datasets of replication cohort. *P < 0.05; **P < 1E-5; ***P < 5E-8

The results of the genetic correlation between T2D and the complications derived from the LDSC and HDL methods were highly consistent and significantly correlated (Fig. 2, Table S2). And there is no significant correlation between dataset pairs in the analysis of DKD, DCM and NAFLD (Fig. 2, Table S2). Additionally, it should be noted that there are results with *rg* greater than 1 in HDL analysis. Here's both the LDSC and HDL developers explain that when the real *rg* is close to the boundary (− 1 or 1) (Table S2), it’s possible that the estimate (true rg + error) will be greater than 1 (Bulik-Sullivan et al. 2015; Ning et al. 2020).

### Primary analysis and colocalization

**Fig. 3:**
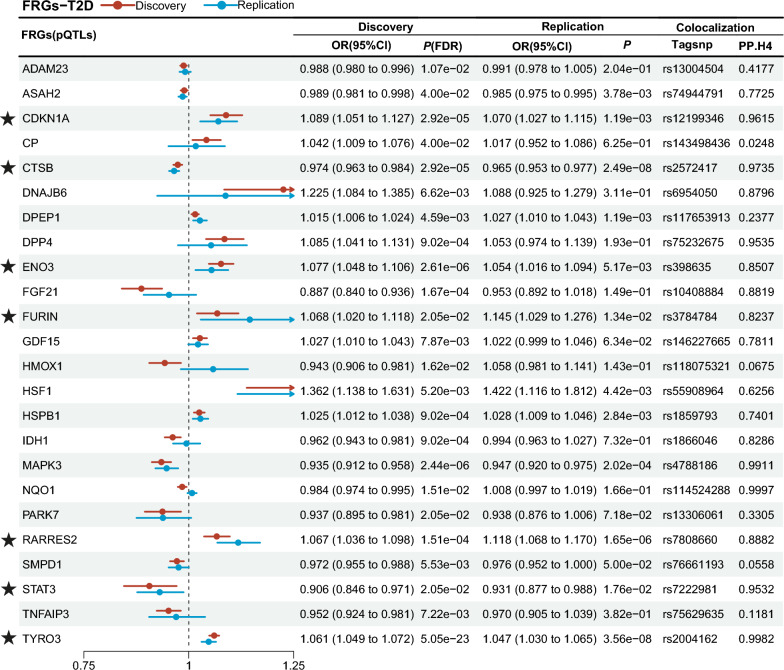
Results of primary analysis and colocalization (pQTLs). FRGs, ferroptosis-related genes; T2D, type 2 diabetes; ★, FRGs on a level of significance of discovery cohort, replication cohort and shared the genetic variations regions (PP.H4 > 0.8)

**Fig. 4:**
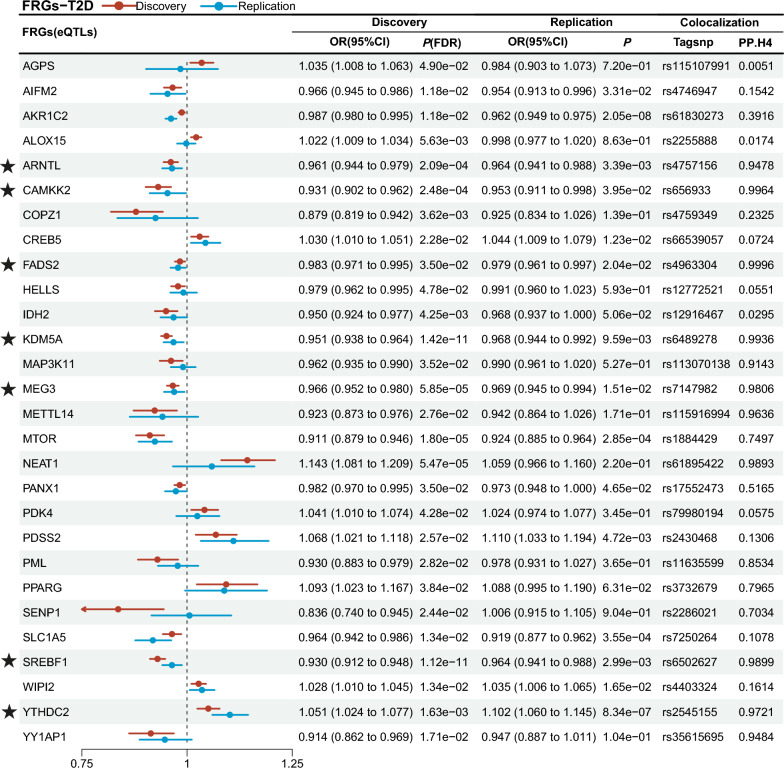
Results of primary analysis and colocalization (eQTLs). FRGs, ferroptosis-related genes; T2D, type 2 diabetes; ★, FRGs on a level of significance of discovery cohort, replication cohort and shared the genetic variations regions (PP.H4 > 0.8)

In the comprehensive analysis of the risk for T2D, FRGs with significant pleiotropy in the discovery cohort or replication cohort were excluded, finally, the expression levels of 14 FRGs were consistently and significantly in the same association direction (Figs. 3, 4, Table S3 to Table S6), along with the colocalization analysis PP.H4 > 0.8 (Tables S7, S8).

Genetically predicted higher levels expression of six genes (*CDKN1A*, *ENO3*, *FURIN*, *RARRES2*, *TYRO3* and *YTHDC2*) were positively associated with T2D risk. Conversely, genetically predicted higher levels expression of *ARNTL, CAMKK2, CTSB, FADS2, KDM5A, MEG3, SREBF1* and *STAT3* were inversely associated with T2D risk. The 14 FRGs will be incorporated into the further analysis of three crucial related complications of T2D. Other FRGs were excluded as they failed to pass all the aforementioned tests.

### Secondary analysis and tissue-specific validation

**Fig. 5:**
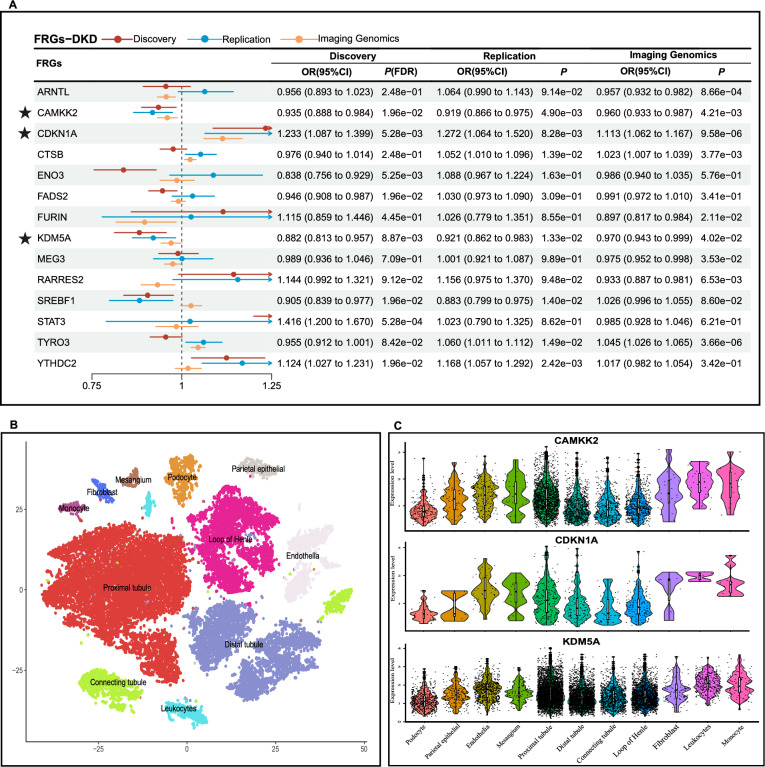
Results of secondary analysis and tissue-specific validation in DKD. **A** Forest plot of discovery cohort, replication cohort, and imaging genomics validation in DKD; **B**11 cell types of kidneys were identified using tSNE clustering; **C** Violin plot of FRGs with significant correlations with DKD. *FRGs* ferroptosis-related genes, *DKD* Diabetic kidney disease; ★, FRGs on a level of significance of discovery cohort, replication cohort and supported by imaging genomics

**Fig. 6:**
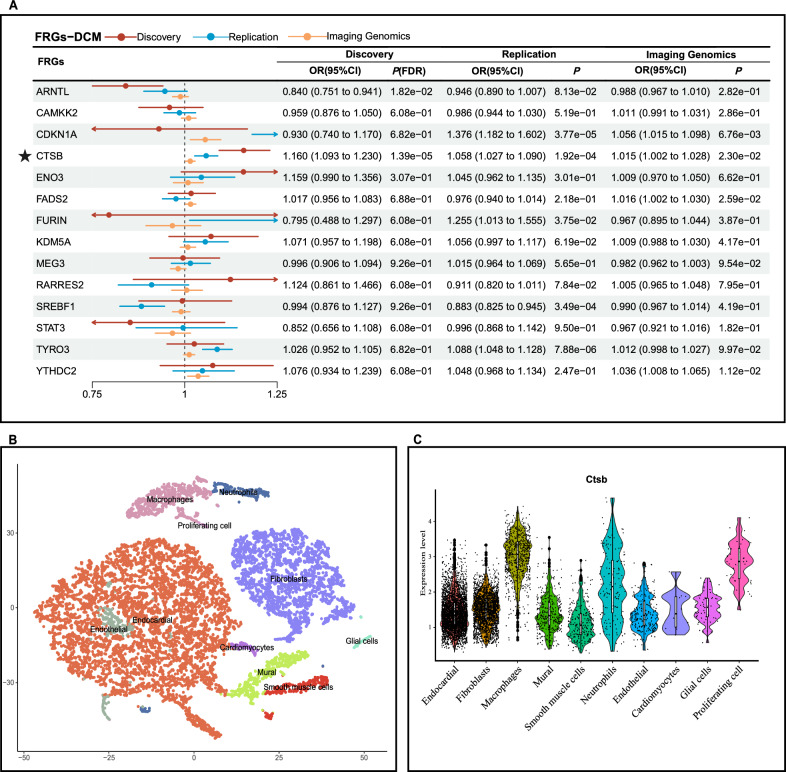
Results of secondary analysis and tissue-specific validation in DCM. **A** Forest plot of discovery cohort, replication cohort, and imaging genomics validation in DCM; **B** 10 cell types of heart were identified using tSNE clustering; **C** Violin plot of FRGs with significant correlations with DCM. FRGs, ferroptosis-related genes; *DCM* Diabetic cardiomyopathy; ★, FRGs on a level of significance of discovery cohort, replication cohort and supported by imaging genomics

**Fig. 7:**
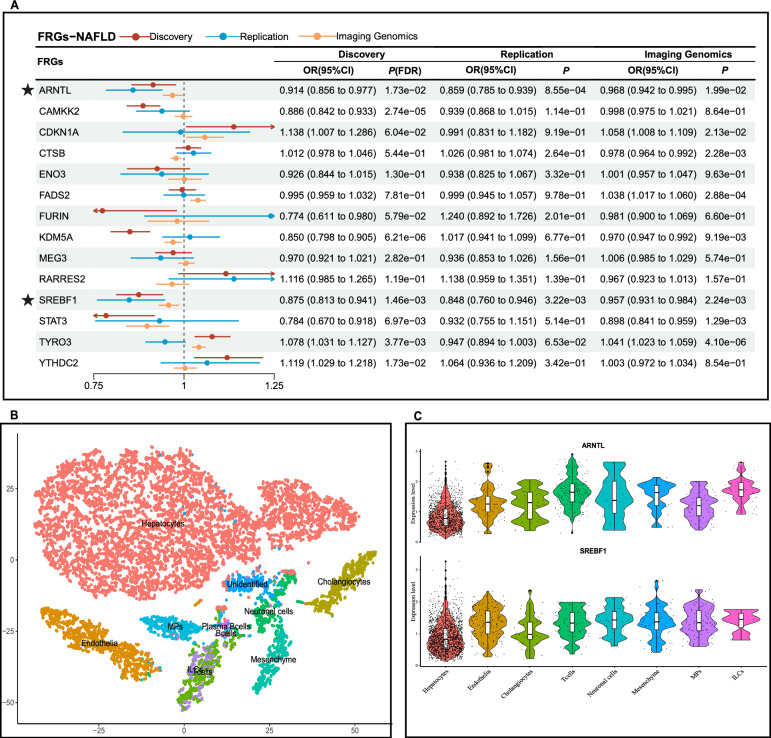
Results of secondary analysis and tissue-specific validation in NAFLD. **A** Forest plot of discovery cohort, replication cohort, and imaging genomics validation in NAFLD; **B** 11 cell types of liver were identified using tSNE clustering; **C** Violin plot of FRGs with significant correlations with NAFLD. *FRGs* ferroptosis-related genes, *NAFLD* Non-alcoholic fatty liver disease; ★, FRGs on a level of significance of discovery cohort, replication cohort and supported by imaging genomics

In the secondary analysis of DKD, three FRGs were fully supported by discovery cohort, replication cohort and imaging genomics. Genetically predicted higher level of *CDKN1A* was positively associated with DKD risk. Genetically predicted higher levels of *CAMKK2* and *KDM5A* were associated with a decreased risk of DKD (Fig. 5A and Table S9). In the analysis of DCM, *CTSB* was the sole one that is fully supported by discovery cohort, replication cohort and imaging genomics (Fig. 6A and Table S10). While genetically predicted higher levels of *CTSB* was positively associated with DCM risk. For NAFLD, genetically predicted higher levels of *ARNTL* and *SREBF1* were associated with a decreased risk of NAFLD and were mutually confirmed in three cohorts (Fig. 7A and Table S11).

The three scRNA datasets were processed as described above, Cells were clustered and annotated into 11 cell types of kidneys (DKD) (Fig. 5B), 10 cell types of heart (DCM, mouse) (Fig. 6B), and 11 cell types of liver (NAFLD) respectively (Fig. 7B). Based on the specific expression of DKD-scRNA, *CAMKK2* and *CDKN1A* were mainly enriched in renal tubules cells, while *KDM5A* was enriched more prominently in all cell types, with relatively higher expression level in proximal tubules, distal tubules, and collecting ducts compared to podocytes and endothelial cells (Fig. 5C and Figure S1). Renal tubules are also the site where ferroptosis and other forms of cell death often occur in DKD. *Ctsb* was mainly enriched in endocardial cells and fibroblasts, where most cell death and fibrosis occur. At the same time, the level of enrichment in macrophages was also higher than that of other cell types (Fig. 6C and Figure S2). As shown in Fig. 7C and Figure S3, for NAFLD, both *ARNTL* and *SREBF1* were predominantly enriched in hepatocytes. While ferroptosis induced by hepatic steatosis and lipid peroxidation may lead to the development of the simple NAFLD to non-alcoholic steatohepatitis (NASH).

## Discussion

In this study, we undertake a comprehensive exploration of the causal correlations between FRGs and T2D as well as its major complications. FRGs that were significantly related to T2D and possessed shared genetic causal variations (PP.H4 > 0.8) were incorporated into the secondary analysis. The functions of FRGs in T2D and significant complications were analyzed from multiple perspectives.

*CAMKK2* (calcium/calmodulin-dependent protein kinase kinase 2) activates AMP-activated protein kinase (*AMPK*) by phosphorylating *AMPK* at the Thr172 site. Such phenomena are typically regarded as secondary markers for diabetes management (Wang et al. 2023). Simultaneously, the attenuated function of *CAMKK2* in endothelial cells might influence transendothelial iron transport and intracellular iron homeostasis by affecting *TFRC* and *TF*, thereby affecting the level of mitochondrial metabolite transporters and endothelial cell bioenergetics (Sabbir 2020). Based on the aforementioned mechanisms, *CAMKK2* has been utilized in some studies related to neurological and mental disorders such as bipolar disorder (Lee et al. 2022), yet its role in kidney diseases remains scarce.

*CDKN1A* (cyclin-dependent kinase inhibitor 1A) gives rise to pre-mRNA composed of three exons and two introns, encoding the p21 protein, and plays an exceedingly crucial role in the p53-mediated cell cycle arrest process (Xiong et al. 1993). This mechanism is in accordance with numerous experiments and studies. Telomere shortening, which limits the proliferative lifespan of human cells, triggers aging phenotypes via the DNA damage pathway, and it can be observed that p21 is significantly upregulated (Choudhury et al. 2007). On the contrary, in the ferroptosis mechanism, *CDKN1A* can restrain the cell cycle process and influence glutathione metabolism, thus occupying an important position in the study of ferroptosis in tumors (Abbas and Dutta 2009). Research has revealed that the ubiquitous p21 deficiency since birth confers glomerular and tubular protection in diabetic mice (Al-Douahji et al. 1999). Previous studies have demonstrated that the level and duration of p21 induction determine the onset of cell growth arrest; (ii) p21 expression is adequate to induce senescence; (iii) p21 expression is restricted to renal tubular cells and is sufficient to induce renal fibrosis after acute kidney injury (Chang et al. 2000).

*KDM5A* is the most abundant member within the *KDM5* family. The inactivation of *KDM5A* mimics the cellular response elicited by hypoxia. *KDM5A* possesses a Jumanji-C (JmjC) domain and is downregulated under hypoxic conditions (Batie et al. 2019). Hypoxia also exerts a significant role in diseases such as cancer, myocardial ischemia, ischemic brain injury, chronic kidney disease, and heart disease (Chen et al. 2020). Recent studies have demonstrated that *KDM5A* might function as an oxygen-sensitive enzyme (Batie et al. 2019). *KDM5A* is a negative regulator of histone *H3K4* trimethylation, while *TGF-β1* induces kidney aging through H3K4me3, ultimately resulting in renal fibrosis and inflammation (Shimoda et al. 2019).

*CTSB* (cathepsin B), a cysteine proteolytic enzyme, is prevalently expressed in diverse cells and predominantly localized within lysosomes. *CTSB* is also implicated in regulating programmed necrotic cell death, pyroptosis, ferroptosis, as well as autophagy (Yadati et al. 2020). It is extensively expressed in the myocardium and participates in the pathophysiological processes of various cardiovascular-related disorders, encompassing atherosclerosis, myocardial infarction, diabetic cardiomyopathy, dilated cardiomyopathy, and even hypertension (Larionov et al. 2019). The mechanism through which *CTSB* engages in ferroptosis might be that lipid peroxidation of the lysosomal membrane during ferroptosis triggers the release of catalytic *CTSB*, which further impairs the mitochondrial membrane and promotes cell death (Nagakannan et al. 2021). *CTSB* is intimately associated with metabolic disorders such as type 2 diabetes, atherosclerosis, cardiac reperfusion injury, and chronic kidney disease through regulating the *NLRP3* inflammasome in the cytoplasm (Yadati et al. 2020).

A notable phenomenon is that its correlation with T2D and that with DCM are not in the same direction. In the analysis process of T2D. By referring to the literature, thus far, there have been no large-scale studies on *CTSB* and T2D, and individual independent studies have presented discrepancies in the conclusions regarding the correlation between *CTSB* and T2D (Hsing et al. 2010; Araujo et al. 2018). The reason could be that *CTSB* varies at different stages of disease progression, or the current sample size is insufficient to fully characterize all the mechanisms of *CTSB* in T2D and related complications.

*ARNTL* (also named *BMAL1*), as a heterodimer formed with Circadian Locomotor Output Cycles Kaput (CLOCK) and *NPAS2*, is a key regulatory molecule of the biological clock gene (Patke et al. 2020). In mammals, the biological clock not only has a significant effect on the behavioural states of animals, but also affects the regulation of many aspects such as neurology, metabolism, endocrinology, cardiovascular and immune functions (Yang et al. 2019). The synchronization of the physiological circadian rhythm is extremely crucial for metabolic health and is also an important strategy for the treatment of obesity and NAFLD (Ray et al. 2020). In this study *ARNTL* was negatively correlated with T2D and NAFLD, the result is consistent with existing studies.

*SREBF1*, the main liver subtypes of *SREBF*, promotes the synthesis of fatty acids and triglycerides in the liver in general. Based on the mechanism of action of *SREBF1*, it appears somewhat contradictory to the analysis result of the negative correlation with NAFLD in this study. However, the underlying mechanism and role of *SREBF1* is not so simple. Studies have shown that even for NAFLD, the expression of *SREBF1* is not consistent (Saran et al. 2020; Pettinelli and Videla 2011). There are also studies have shown that *SREBF1* could regulating lipid synthesis and degradation in the liver by completely different mechanism (Nguyen et al. 2021). To further validate the specific expression of SREBF1 in the liver, subcutaneous adipose, and visceral adipose, we intended to derived relevant expression data from GTEx. Nevertheless, the most significant SNPs of the tissue-specific eQTLs of these three tissues were rs57886565 (*P* = 6.14E-05), rs113029629 (*P* = 1.94E-05), and rs117107422 (*P* = 2.47E-05) respectively (GTEx Consortium 2020), and therefore the MR or SMR analysis of this study could not be accomplished. In addition, reports on the regulation of *SREBF1* mainly focus on its protein or post-translational level and may be regulated by epigenetics. Larger sample-sized eQTLs or pQTLs studies are needed for further verification.

We observe that in numerous studies concerning ferroptosis and other mechanisms, targeted therapeutics, including drug development, have primarily focused on anti-tumor. Nevertheless, as complications of T2D, such as DKD, DCM, and NAFLD are non-tumor disorders, the mechanism of ferroptosis in these diseases could be diametrically opposite. For instance, several *KDM5A* inhibitors have demonstrated promising outcomes in anti-cancer (Vinogradova et al. 2016), and there is also a *KDM5A* inhibitor, GS-5801, which has entered phase 1 clinical trials for chronic hepatitis B (Gilmore et al. 2017). Through the analysis results of this study, it indicates us that attention must be paid to kidney or liver damage and related adverse reactions during the application process, especially when the patient has a history of T2D. Another example is that there are numerous drugs targeting *SREBF1*, many of which are utilized in lipid lowering, treating NAFLD or in tumor research (Cai et al. 2023), as well as being newly discovered targets of some traditional drugs (Deng et al. 2021), etc. And through the analysis results of this study, it is obvious that the tissue-specific expression of *SREBF1* varies significantly, and even in different phases of the progression of T2D and NAFLD, the expression and mechanism of action are dissimilar. Therefore, if the aforementioned drugs are applied clinically, the indicators that need to be closely monitored in different phases of the disease should be further investigated.

## Advantage

(i) In this study, imaging genomics was considered as a powerful reference for multi-perspective verification. From another perspective, verification for imaging genomics was provided through the discovery cohort and replication cohort, thereby offering a novel non-invasive examination strategy for the diagnosis, treatment, and prognosis of T2D’s complications. (ii) A systematic combinatorial analysis of extensive data on a large number of T2D cases, encompassing genomics, transcriptomics, proteomics, and imaging genomics, was carried out to explore the association between FRGs and T2D and its significant complications. (iii) A meta-analysis of large datasets of eQTLs and pQTLs was conducted to enhance statistical power and evade bias.

## Limitations

Our research results may be influenced by the following potential limitations: (i) Cell death and ferroptosis are only part of the underlying mechanisms of T2D and related complications. This study takes an in-depth look at ferroptosis, which may overlook the universality of T2D biomarkers and diagnosis. Additionally, there is crosstalk and overlap between ferroptosis and other cell death modalities, and there may be significantly related ferroptosis-related genes (FRGs) that are not primarily involved in the pathogenesis of ferroptosis. The specific mechanisms need to be further verified through experiments. (ii) It is impossible to completely distinguish the complications caused by T1D and T2D; (iii) Due to the fact that most of the data in this analysis are from the European population, the generalization of the analysis is limited. (iv) In addition, given that the mechanism of the disease encompasses multilayer regulations, including DNA methylation, splicing, and post-translational modification (PTM), the risk prediction and drug development of identified FRGs are also worthy of in-depth exploration. (v) The correlation and differences in the impact of circulating proteins and tissue proteins on diseases need to be further validated through experiments and cohort studies. (vi) Additional and refined single-cell sequencing data from models of T2D and related complications will help validate the expression and mechanisms of action of ferroptosis-related genes in tissues.

## Conclusions

Our research, supported by multi-omics evidence, identified several ferroptosis-related genes (FRGs) associated with the risk of T2D and its complications. Additionally, both the discovery and replication cohorts, along with other omics data, provided validation for the use of imaging genomics in non-invasive diagnosis and prognostic assessment of the disease. However, further experiments, clinical cohort studies, and medical imaging research are necessary to evaluate the practicality and effectiveness of these candidate genes.

## Supplementary Information

Supplementary material 1.

## Data Availability

No datasets were generated or analysed during the current study.

## Abbreviations

T2D: Type 2 diabetes
DKD: Diabetic kidney disease
ESRD: End-stage renal disease
DCM: Diabetic cardiomyopathy
NAFLD: Non-alcoholic fatty liver disease
NASH: Non-alcoholic steatohepatitis
ROS: Reactive oxygen species
FRGs: Ferroptosis-related genes
MR: Mendelian randomization
GWAS: Genome-wide association studies
LDSC: Linkage disequilibrium score
HDL: High-definition likelihood
FDR: False discovery rate
IVW: Inverse-variance weighted
pQTL: Protein quantitative trait loci
eQTL: Expression quantitative trait loci
scRNA: Single-cell RNA sequencing
OR: Odds ratio
CI: Confidence interval
