# Supplementary figures and images for: Uncovering the molecular networks of ferroptosis in the pathogenesis of type 2 diabetes and its complications: a multi-omics investigation
Source: Mol Med. 2024 Dec 23;30:268. doi: 10.1186/s10020-024-01045-w (PMC11665190; doi:10.1186/s10020-024-01045-w)

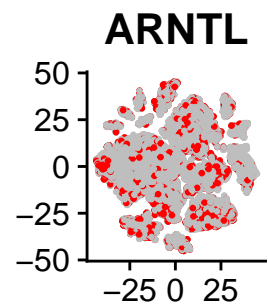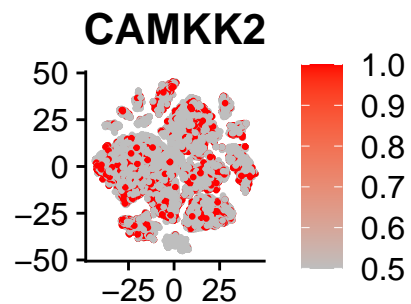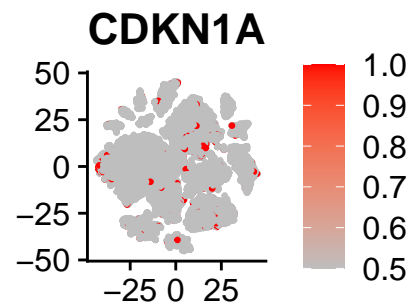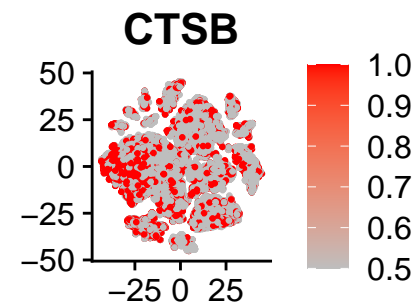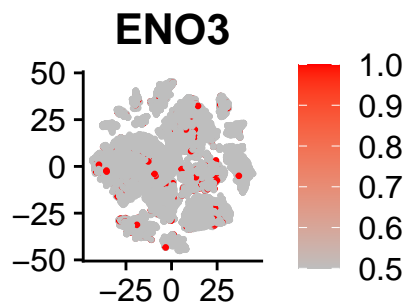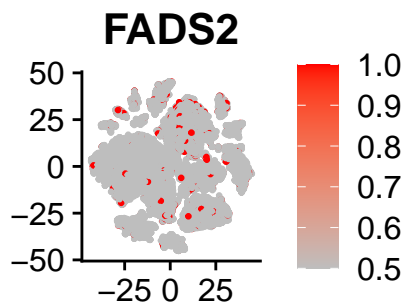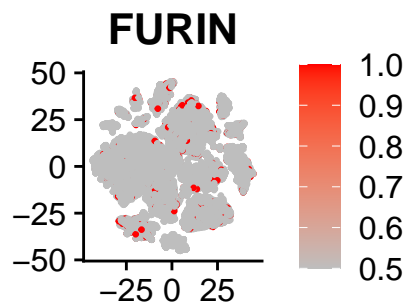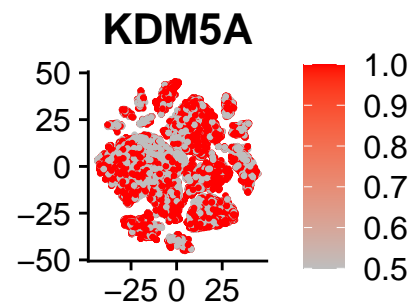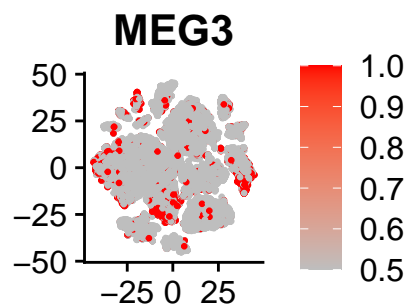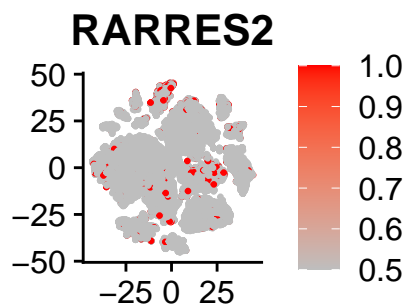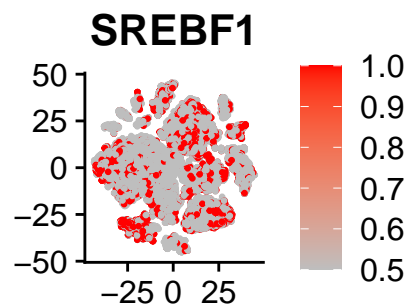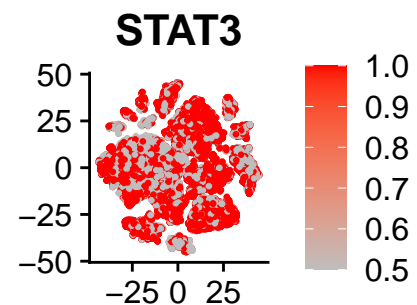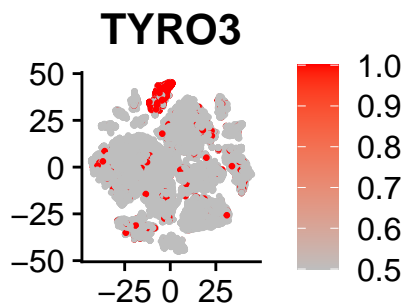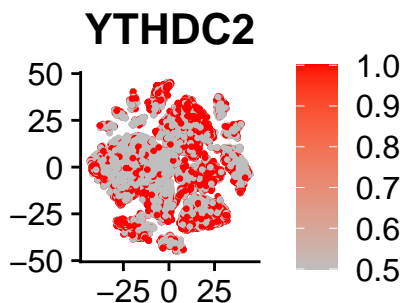

Supplement: Supplementary file 1 — Supplementary material 1. [file 10020_2024_1045_MOESM1_ESM.zip › New folder/Figure S1.pdf]

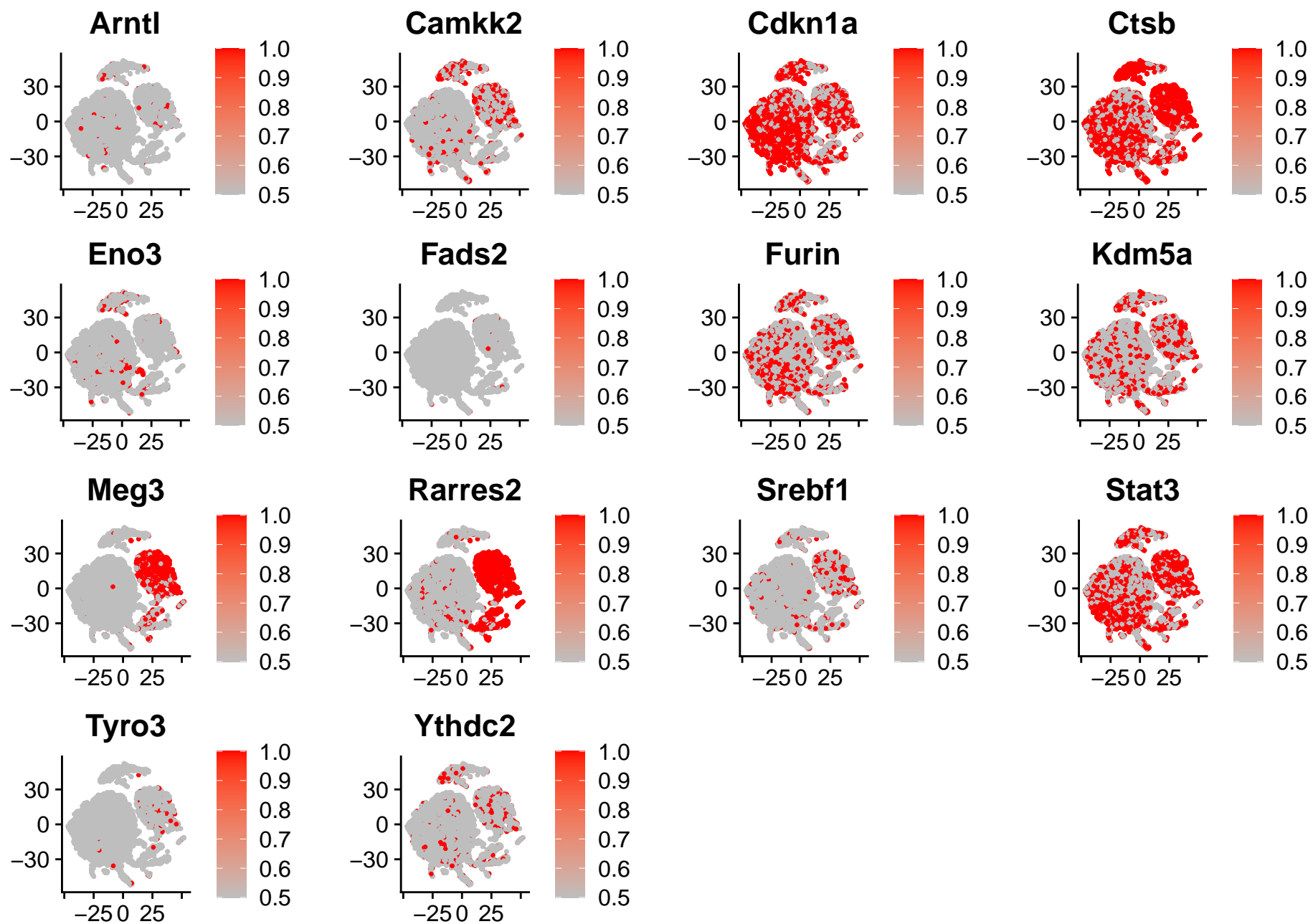

Supplement: Supplementary file 1 — Supplementary material 1. [file 10020_2024_1045_MOESM1_ESM.zip › New folder/Figure S2.pdf]

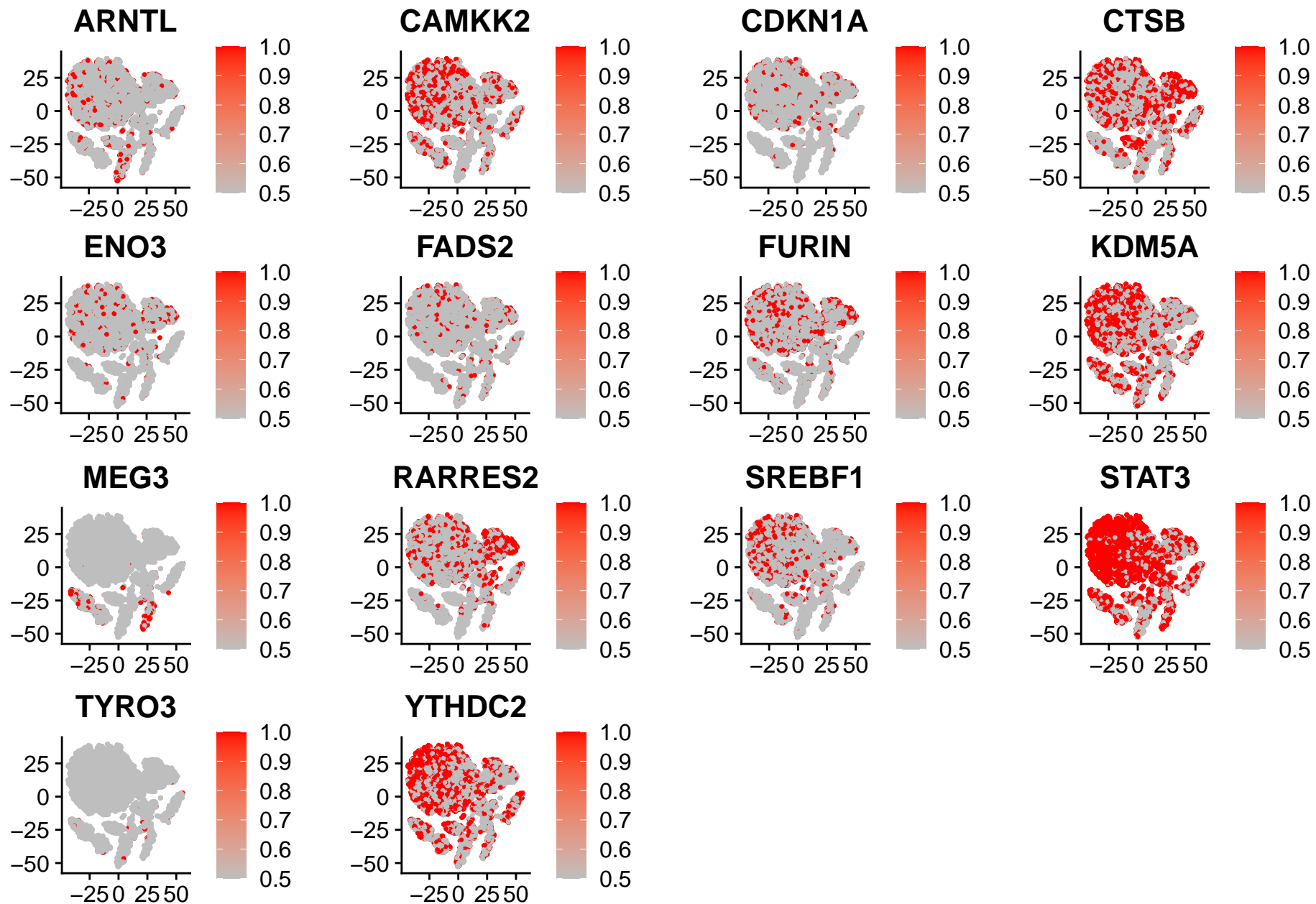

Supplement: Supplementary file 1 — Supplementary material 1. [file 10020_2024_1045_MOESM1_ESM.zip › New folder/Figure S3.pdf]
